# Different Patterns of Evolution in the Centromeric and Telomeric Regions of Group *A* and *B* Haplotypes of the Human Killer Cell Ig-Like Receptor Locus

**DOI:** 10.1371/journal.pone.0015115

**Published:** 2010-12-29

**Authors:** Chul-Woo Pyo, Lisbeth A. Guethlein, Quyen Vu, Ruihan Wang, Laurent Abi-Rached, Paul J. Norman, Steven G. E. Marsh, Jeffrey S. Miller, Peter Parham, Daniel E. Geraghty

**Affiliations:** 1 Clinical Research Division, Fred Hutchinson Cancer Research Center, Seattle, Washington, United States of America; 2 Department of Structural Biology, Stanford University School of Medicine, Stanford, California, United States of America; 3 Anthony Nolan Research Institute, London, United Kingdom; 4 University of Minnesota, Minneapolis, Minnesota, United States of America; Duke University, United States of America

## Abstract

The fast evolving human *KIR* gene family encodes variable lymphocyte receptors specific for polymorphic HLA class I determinants. Nucleotide sequences for 24 representative human *KIR* haplotypes were determined. With three previously defined haplotypes, this gave a set of 12 group *A* and 15 group *B* haplotypes for assessment of *KIR* variation. The seven gene-content haplotypes are all combinations of four centromeric and two telomeric motifs. *2DL5*, *2DS5* and *2DS3* can be present in centromeric and telomeric locations. With one exception, haplotypes having identical gene content differed in their combinations of *KIR* alleles. Sequence diversity varied between haplotype groups and between centromeric and telomeric halves of the *KIR* locus. The most variable *A* haplotype genes are in the telomeric half, whereas the most variable genes characterizing *B* haplotypes are in the centromeric half. Of the highly polymorphic genes, only the *3DL3* framework gene exhibits a similar diversity when carried by *A* and *B* haplotypes. Phylogenetic analysis and divergence time estimates, point to the centromeric gene-content motifs that distinguish *A* and *B* haplotypes having emerged ∼6 million years ago, contemporaneously with the separation of human and chimpanzee ancestors. In contrast, the telomeric motifs that distinguish *A* and *B* haplotypes emerged more recently, ∼1.7 million years ago, before the emergence of *Homo sapiens*. Thus the centromeric and telomeric motifs that typify *A* and *B* haplotypes have likely been present throughout human evolution. The results suggest the common ancestor of *A* and *B* haplotypes combined a *B*-like centromeric region with an *A*-like telomeric region.

## Introduction

Humans, apes and monkeys have expanded families of genes encoding killer cell immunoglobulin-like receptors (KIR) (reviewed in [1]). Through the recognition of MHC class I, KIR regulate the development and response of natural killer (NK) cells (reviewed in [2]). KIR are also expressed by subpopulations of αβ and γδ T cells [3]. In their function, genetics and variegated expression, primate KIR are very similar to rodent Ly49, although these common properties are the result of convergent evolution [4]. Because both receptor and ligand are highly polymorphic, the interactions between KIR and HLA class I that regulate NK cell function are extraordinarily diverse. And this is further increased by the influence of variable peptides that bind to HLA class I and are contacted by KIR [5], [6]. As a consequence, variability in KIR genotype in human populations is associated with as wide-ranging a collection of diseases as HLA. Notably, they include susceptibility to infection [7], [8] and autoimmunity [9], [10], [11], [12], [13], [14], [15], the outcome of hematopoietic cell transplantation [16], [17] and the success of placental reproduction [18].

Genetic diversity in the human *KIR* gene family arises from two factors: variability in *KIR* gene content and allelic polymorphism [19]. Because of the propensity for asymmetric recombination within the *KIR* gene family, the simple definitions of genes and alleles do not always apply [20], and as a consequence the reported number of human *KIR* genes varies. By conservative account, the human *KIR* family consists of 11 genes (*2DL1*, *2*/*3*, *4* and *5*; *2DS1*, *2*, *4* and *3*/*5*; and *3DL1*/*S1*, *3DL2* and *3*) and two pseudogenes (*2DP1* and *3DP1*). Through the combination of gene-content diversity and allelic polymorphism, the variability in *KIR* genotype is such that most pairs of unrelated human individuals have different KIR genotypes [21], as is also the case for HLA class I [22]. A unique feature of the human *KIR* system, and one not mirrored in other higher primates, is the segregation of two distinctive groups of haplotypes (*A* and *B*) [23], which are present in all the >150 human populations examined and are maintained by balancing selection [24]. The group *A* haplotypes have a simple and constant gene content, dominated by genes encoding inhibitory receptors. In contrast, the group *B* haplotypes have variable and greater gene content, involving genes encoding distinctive inhibitory receptors and a variety of activating receptors [23].

Although the structures of numerous *KIR* haplotypes have been indirectly inferred and deduced from population analysis and family studies [19], [25], [26], [27], [28], [29], [30], [31], complete determination of human KIR haplotype structures using direct methods has so far been limited to one *A* haplotype and two *B* haplotypes [25], [32], [33]. Given the decisive role of KIR variability in modulating the human NK cell response during infection, pregnancy and allogeneic transplantation, it became essential that the structures of the common *KIR* haplotypes be unambiguously defined. To this end we determined the sequences of a balanced selection of 24 common group *A* and *B KIR* haplotypes.

## Results

**Figure 1:**
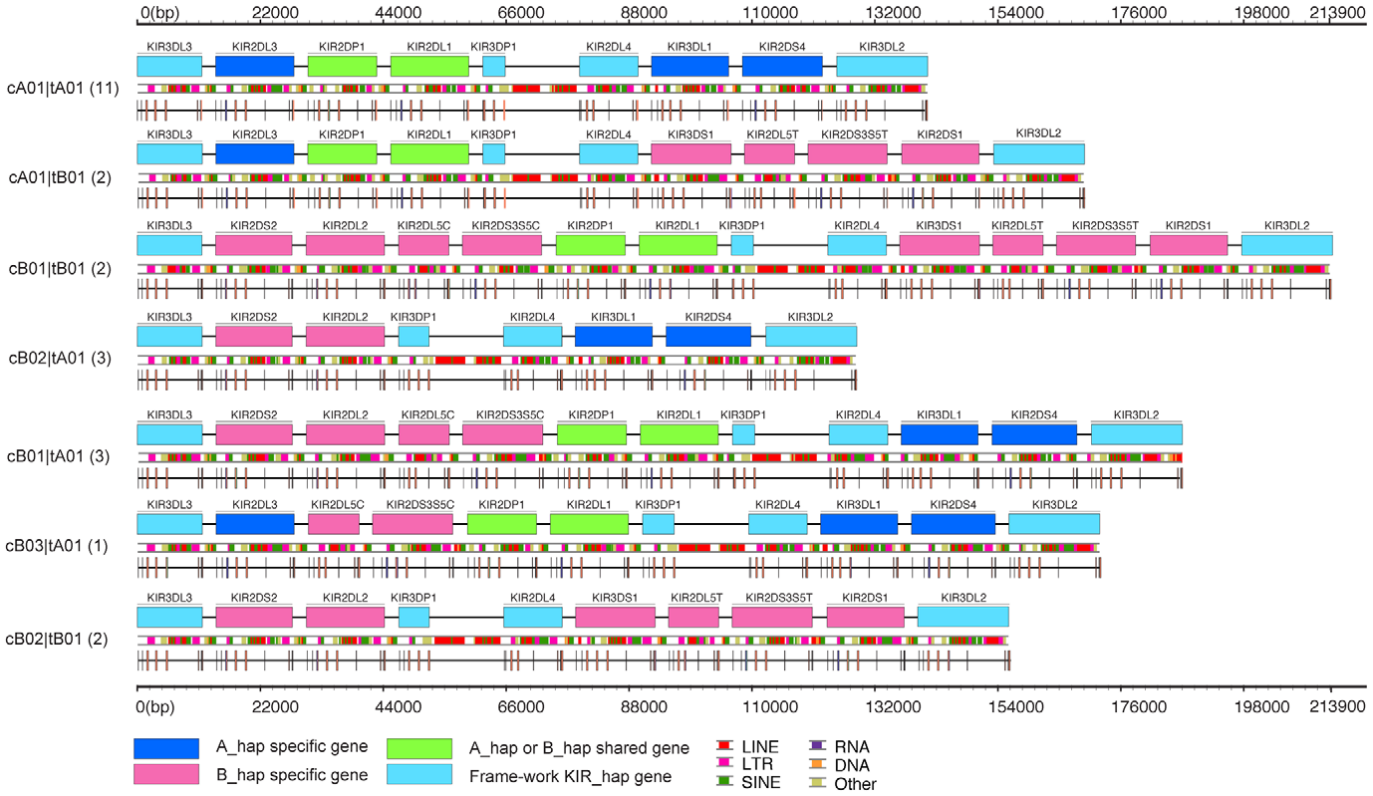
Genomic sequencing of seven gene-content *KIR* haplotypes represented in a panel of 11 *A* and 13 *B KIR* sequences. KIR gene content haplotype structures found from the phased sequences of 24 haplotypes. Each haplotype is named according to the WHO nomenclature as indicated to the left of each sequence, with the numbers of each haplotype resequenced indicated in parenthesis. The key at the bottom designates the color-coding used to distinguish the genes according to type, and the repeat sequence structures found within each sequence. The structure for each gene and pseudogene is indicated beneath with bars indicating exons.

Based upon *KIR* gene content, cell lines derived from 12 individuals were chosen for complete sequence analysis of *KIR* haplotypes. In choosing these cells, two selection criteria were applied: first, that each cell line was inferred to carry both an *A* and *B KIR* haplotype; second, that all common gene haplotypes in the *B* group were represented in the panel of cell lines. As predicted, eleven members of the panel had both an *A* and *B* KIR haplotype. The twelfth member of the panel (from cell line GRC212) had two *B* haplotypes, one sharing the centromeric part of the locus with the *A* haplotype and the other sharing the telomeric part with *A*. Combining these haplotypes with three other *KIR* haplotypes deposited in Gen-Bank [32], [33], [34] gave a data set of 27 *KIR* haplotypes in which seven gene-content haplotypes were represented (Fig. 1). Twelve haplotypes are of group *A*, and 15 of group *B*. Four of the six group *B* gene-content haplotypes were represented more than once. Common to the 27 haplotypes were the three framework regions of the KIR locus originally defined by Trowsdale and colleagues [33]: *KIR3DL3* at the centromeric end, *KIR3DL2* at the telomeric end, and the combination of *KIR3DP1* and *KIR2DL4* in the middle of the locus.

In order to determine the representative frequency of each of the sequenced haplotypes, including an assessment of how inclusive our sequenced haplotypes are of those common in populations, we examined a panel of 192 DNAs including 48 each of Caucasian, Asian, African-American, and Hispanic. Using a panel of STS assays specific for each of the KIR genes and pseudogenes as described in Methods we were able to provide a total gene content analysis for each individual. A subset of these assays included PCR primers that extended from the end of one gene to the start of an adjacent gene allowing us to determine *cis* and *trans* relationships among some pairs of genes. These data altogether allowed us to deduce the pair of haplotypes contained within each individual, with the exceptions of two pairs of ambiguities - cA01|tA01-cB01|tB01 versus cA01|tB01-cB01|tA01 and cA01|tA01-cB02|tB01 versus cA01|tB01-cB02|tA01. We used estimated frequencies of each of these haplotypes calculated by linkage disequilibrium estimates of the haplotype frequencies to adjust for these ambiguities. Of the 7 haplotypes sequenced, 6 were relatively common in all populations examined while the seventh was found in only 2 individuals of African descent (Table 1). In addition, a total of 21 chromosomes did not match any of the sequenced haplotype patterns, including 13 chromosomes in the African-American samples. However, of the 12 distinct haplotypes that made up this group, none were present at more than 1% of the total. We did not fully characterize these haplotypes other than to exclude them from among the 7 sequenced haplotypes and to distinguish them among themselves and presume they may include examples of the reported rare KIR haplotypes [20], [35], [36].

**Table 1:** KIR haplotype frequencies in 192 reference samples.

KIR Haplotype	AFA n = 96	ASI n = 96	HIS n = 96	CAU n = 96	Total n = 384 (%)
**cA01|tA01**	52	61	59	60	232 (60.4)
**cA01|tB01**	5	15	15	9	45 (11.7)
**cB01|tB01**	2	1	1	6	10 (2.6)
**cB02|tA01**	6	5	8	15	34 (8.9)
**cB01|tA01**	14	2	8	4	28 (7.3)
**cB02|tB01**	2	6	4	1	13 (3.4)
**cB03|tA01**	2	0	0	0	2 (0.5)
**other**	13	6	1	1	21 (5.5)

### A diversity of *KIR* haplotypes is formed from few centromeric and telomeric gene content motifs

Dividing the *KIR* haplotypes into centromeric and telomeric regions separated by the *3DP1-2DL4* framework [33], showed that the seven gene content *KIR* haplotypes are all combinations of four centromeric and two telomeric gene-content motifs (Figure 1). Motif *Cen-B3* is present on a single haplotype, whereas the other motifs are represented on 5 to 19 haplotypes (Table 2). Excluding *Cen-B3*, all possible combinations of the remaining three centromeric and two telomeric motifs are represented in the 27 KIR haplotypes. Because much of KIR diversity arises from recombinatorial association of centromeric and telomeric motifs, a logical and adaptable nomenclature for haplotypes has been based on this principle and is introduced in Figure 1.

**Table 2:** KIR motif frequencies.

MOTIF		Number of allele motifs	Number of haplotypes
Cen	**A1**	13	14
	**B1**	4	6
	**B2**	6	6
	**B3**	1	1
Tel	**A1**	13	19
	**B1**	4	8

**Figure 2:**
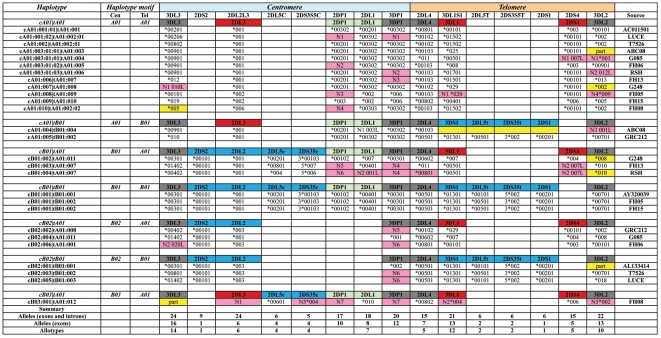
Four centromeric and two telomeric gene-content motifs combined with allelic variation diversifies *KIR* haplotypes. The names of the centromeric (*Cen*) and telomeric (*Tel*) motifs are given at the left under the heading ‘Haplotype’. The structures of each of seven gene content haplotypes are indicated above each group of sequenced haplotypes that contained the respective motif. Gene names are given in boxes: colored gray for framework genes, red for *Cen-A* and *Tel-A* genes, blue for *Cen-B* and *Tel-B* genes, and green for genes found in both *Cen-A* and *Cen-B*. The gene 3LD1S1 is distinguished within each haplotype according to its respective motif residence as red or blue as are 2DS1 and 2DS4. Beneath each gene name are listed the respective allele number within each sequenced haplotype with pink color indicating newly discovered alleles. Yellow shading indicates that the gene/allele sequence was incomplete. Shown to the right is the source, either the cell line name or GenBank entry, for each haplotype. The haplotype names for each sequenced haplotype are based on the allele combination in the centromeric and telomeric gene motifs. For example, the haplotype AC011501 at the top of the table has the *Cen-A1* and *Tel-A1* motifs. It was assigned 001 for each of the allelic combinations comprising these motifs and has thus been designated *cA01:001|tA01:001*. Only two of the 27 haplotypes share identical allele content (*cB01:001|tB01:002*). Under the chart of haplotypes, the numbers of different alleles and allotypes for each *KIR* gene are given.

Although the twelve group *A* haplotypes have identical *KIR* gene content, they all have different combinations of alleles for the constituent *A* haplotype genes (Figure 2). This demonstrates the extent to which allelic polymorphism diversifies group A haplotypes. Allelic polymorphism is also seen to distinguish group *B* haplotypes of identical gene content. However, two haplotypes having the identical *Cen-B1* and *Tel-B1* gene content motif also have identical combinations of *KIR* alleles.

Most polymorphic of the motifs is *Tel-A* with twelve allotype combinations, followed by *Cen-A* with ten allotype combinations. There is considerably less variation in the motifs that define *B* haplotypes, the most diverse being *Cen-B2* with six allele combinations. This hierarchy of allelic variation agrees with that observed in population studies [27], [31]. Fifteen new alleles encoding ten new proteins and fourteen new pseudogene alleles were defined in this study (Fig. 2). All but one of the new variants (2DS5*004) were either alleles of framework genes or genes characteristic of the *A* haplotypes. By applying a similar hierarchical approach to that used for HLA nomenclature [37], the proposed *KIR* haplotype nomenclature can accommodate the higher resolution achieved with the combination of allele sequences, as shown in Figure 2.

### Nucleotide diversity of the KIR region varies with haplotype groups and the two halves of the *KIR* locus

**Figure 3:**
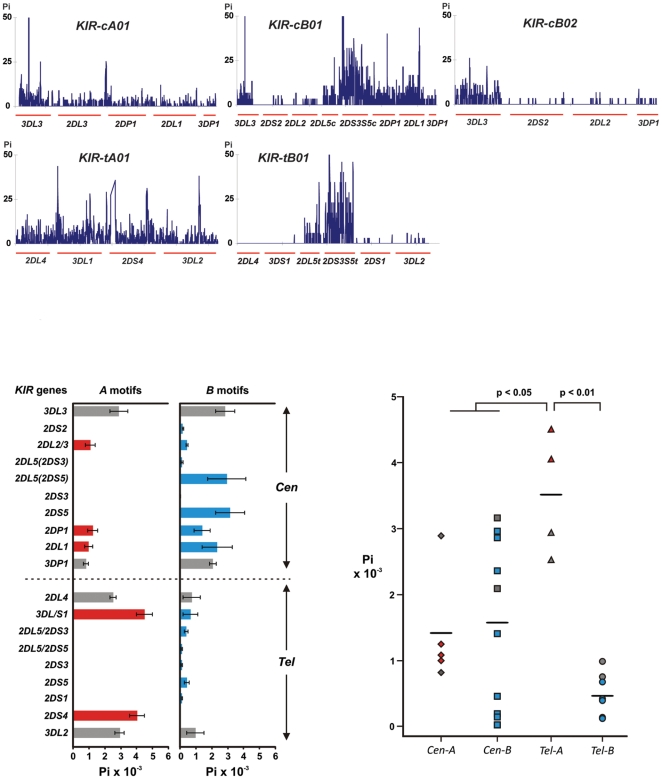
*Tel-A* genes exhibit the highest level of nucleotide variation. Nucleotide diversity, Pi, was calculated for each genomic segment and for each locus separately. Alignment of *KIR* sequences comprising the centromeric and telomeric regions subdivided into 5 of the 6 motifs was examined. *KIR* genes were also examined individually and alleles for framework genes were sorted according to their location on *A* or *B* motifs. *KIR2DL1* and *2DP1* alleles were similarly analyzed according to their presence on *A* or *B* motifs. *KIR2DL5* alleles were subdivided according to their linkage with either *2DS3* or *2DS5*. (A) Shows plots the Pi values over each genomic region for each of the 5 motifs as indicated. (B) Shows the Pi values for the individual *KIR* genes. Characteristic genes of the *A* haplotype are shown in red, characteristic genes of the *B* haplotype are shown in blue, and framework genes are shown in grey. Overall the *Tel-A* genes have the highest diversity. Certain *Cen-B* genes exhibit moderate diversity, with lowest diversity evident for the *Tel-B* genes. (C) Individual data points are the Pi values for each of the genes of *Cen-A*, *Cen-B*, *Tel-A*, and *Tel-B* segments and their mean values (horizontal lines). Pi values for *Cen-A* genes are indicated by diamonds, *Cen-B* by squares, *Tel-A* by triangles, and *Tel-B* by circles. Color coding is the same as in panel B.

To first assess the diversity of the KIR region as a whole, we calculated the nucleotide diversity (Pi) by applying DnaSP [38] to an alignment of *KIR* sequences including all of the sequence data comprising the centromeric and telomeric regions subdivided into 5 of the 6 motifs, each compared separately (Figure 3A, File S1). The cB03 motif was not included at this level of analysis since only one copy was available. Two distinguishing features are the extremely low levels of diversity over *2DL4* and *3DS1* in the KIR-tB01 segment and the substantially higher diversity in the *2DP1-2DL1* segment in *KIR-cB01* versus *KIR-cA01*. Most of the diversity found spanning the 2DS3S5 locus results from the differences between the 2DS3 and 2DS5 alleles and therefore they were analyzed separately in the individual gene analysis immediately following.

To assess the allelic variation of individual *KIR* genes we similarly calculated their nucleotide diversity to an alignment of *KIR* sequences individually, each beginning 250 bp upstream of the start codon and extends throughout the gene to end at the polyadenylation site. In this alignment, the *3DP1* sequences naturally terminate at the end of exon 5. Comparison of variants characterizing the *A* and *B* haplotypes was performed (Figure 3B, C). Thus, subgroups of the framework genes, *2DL1* and *2DP1* were analyzed separately according to their presence on either the *A* or *B* haplotypes. In addition, the *2DL5* sequences were subdivided according to their linkage, either to *2DS3* or *2DS5*, as well as their presence in either the centromeric or telomeric part of the locus (Figure 3B). Of the eight genes and KIR3DP1 common to *A* and *B* haplotypes, only *3DL3* and *2DP1* exhibited similar variability in the two haplotype groups. In contrast, *2DL1* and *3DP1* are more variable in the *B* haplotypes and the other five genes are more variable in the *A* haplotypes. Further distinguishing the haplotype groups, the most variable *A* haplotype genes (*3DL1* and *2DS4*) are in the telomeric part of the locus, whereas the most variable B haplotype genes (*2DL5* and *2DS5*) are in the centromeric part.

The overall diversity in the centromeric and telomeric parts of the *A* and *B* haplotypes was assessed from averages obtained from the Pi values for their constituent genes (Figure 3C). The *3DL1* and *2DS4* genes of the *Tel-A* segments have the highest Pi, a diversity that extends to the neighboring framework genes and makes *Tel-A* significantly more diverse than the other three segments. Although the average nucleotide diversity for *Cen-A* and *Cen-B* is similar, the *Cen-B* segment genes form a bimodal distribution in which *2DS2*, *2DL2L3*, and *2DS3S5* have little diversity, whereas *2DL1*, *2DS5*, and *2DL5* have substantial diversity. Apart from *3DL3*, the *Cen-A* segment genes are of low diversity, which falls between the values for the two groups of *Cen-B* genes. This pattern of variation points to the minimally diverse *Cen-B* genes having either been more recently formed or more recently subject to selection.

**Figure 4:**
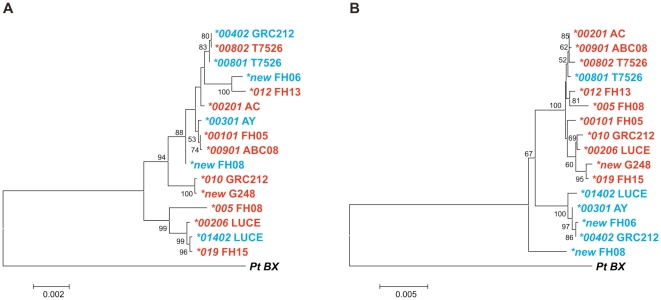
A recombination point in 3DL3 shuffles Ig domain encoding exons with different tail-encoding exons. (A) Shows the NJ trees for the 5′ region of *3DL3* beginning 250 bp 5′ of the start codon and ending 1 kb 3′ of exon 7 (encoding the transmembrane domain). Sequence names are colored according to their presence on *Cen-A* or *Cen-B* segments. (B) Shows the NJ trees for the 3′ region of *3DL3* beginning 1 kb 5′ of exon 7 and ending at the polyadenylation signal. Color coding is as in panel A. The alleles divide according to their presence on *Cen-A* or *Cen-B* segments for the 3′ region and are mixed in the 5′ region. The exception (3DL3*0801 found on a *Cen-B* segment) is a result of a recombination that occurred 3′ of the *3DL3* gene in the intergenic region between it and *2DS2*.

To investigate further the observation that *3DL3* is highly polymorphic in both *Cen-A* and *Cen-B*, we performed domain-by-domain phylogenetic analysis of the alleles of this gene (Figure 4). The analysis revealed a recombination break point between the exons encoding the D2 domain and the transmembrane region. This point of recombination defines two KIR3DL3 lineages that differ in their transmembrane domains and cytoplasmic tails. One of the 3DL3 lineages is exclusively associated with *Cen-A*, whereas the other is exclusively associated with *Cen-B*. The recombination breakpoint also defines two lineages for the extracellular domains of KIR3DL3, but these do not segregate with *Cen-A* and *Cen-B* group but are evenly distributed between them. However, no *3DL3* allele is common to *Cen-A* and *Cen-B*. One exception (3DL3*0801 found on a *Cen-B* segment) appears to be the result of a recombination that occurred 3′ of the *3DL3* gene in the intergenic region between it and *2DS2.*

### *KIR2DS3*, *2DS5*, (*2DS3S5*) and *2DL5* can be present in the centromeric and telomeric parts of the *KIR* locus

Previous studies have shown that two forms of *KIR2DL5* locate to the centromeric (*KIR2DL5B*) and telomeric (*KIR2DL5A*) parts of the *KIR* gene locus [39]. Based upon linkage disequilibrium with *2DL5B* or *2DL5A*, it has also been suggested that *2DS3* and *2DS5* can similarly be present in either centromeric or telomeric locations [31], [40], [41]. The structures of *KIR* haplotypes defined here prove this hypothesis to be true. The centromeric motifs of seven haplotypes contain *2DL5B*, which can associate with 2DS3*00103 or one of three *2DS5* alleles (*new, *005 or *006). Likewise, the telomeric motifs of seven haplotypes contain *2DL5A*, which can associate with either 2DS3*002 or 2DS5*002. Thus *2DL5A* and *2DL5B* both associate with *2DS3* and *2DS5*. In all combinations, the allele of *2DL5* uniquely defines the associated *2DS3* or *2DS5* allele, and vice versa. Particularly variable are the three pairs of associated *2DL5B* and *2DS5* alleles (Fig.3A).

**Figure 5:**
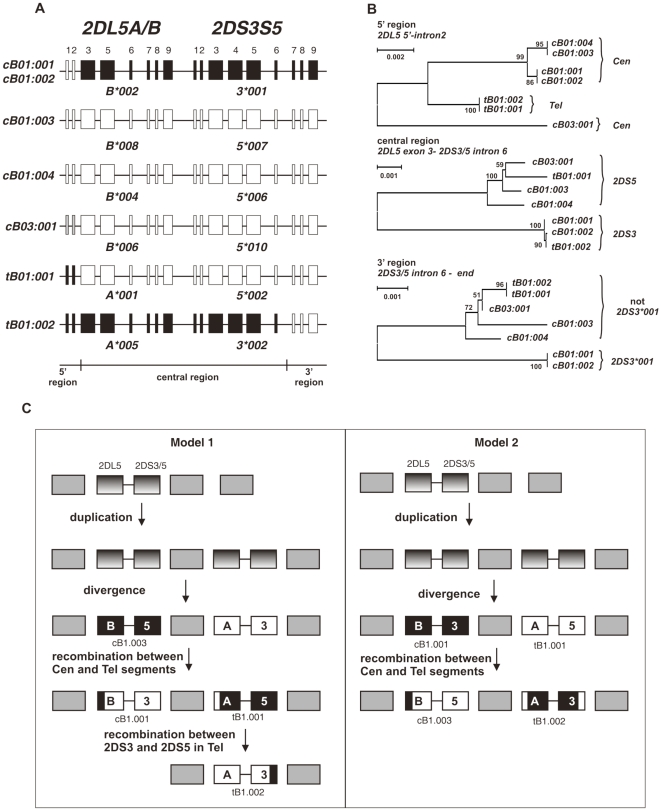
*2DS3* and *2DS5* descend from a common ancestor and diversified by interlocus recombination. (A) Shows the six combinations of *2DL5* and *2DS3/5* present in the 27 haplotypes, along with the motif to which they belong. Shading denotes their relatedness as determined by the phylogenetic analyses shown in panel B. (B) Genomic sequences extending from 5′ of *2DL5* through the end of the neighboring *2DS3/5* gene were aligned, divided into three regions (5′, central, and 3′) and submitted to phylogenetic analysis. Neighbor joining trees are shown, with Bootstrap values for the nodes. For the 5′ region (from 250 bp 5′ of *2DL5* exon 1 through to the end of intron 2), the variants divide according to their location in the centromeric or telomeric half of the *KIR* locus. The central region (from exon 3 of *2DL5* to 1.9 kb 5′ of exon 6 in *2DS3S5*) divides according to association with *2DS3* or *2DS5*. In the 3′ segment (from intron 6 to the polyadenylation signal at the end of *2DS3S5*) the variants containing 2DS3*001 form an outgroup. (C) Shows two models for the evolution of *2DS3* and *2DS5*. Both models start with a single *2DL5A/B-2DS3/5* progenitor in the centromeric region, the only site where chimpanzee and orangutan 2DL5 has been found ([65] and AC220148). This progenitor then duplicated, with one daughter being transposed to the telomeric region. Subsequent diversification at the two loci resulted in distinct *2DL5A*, *2DL5B*, *2DS3* and *2DS5* genes. The two models differ in the sites where *2DS3* and *2DS5* arose: in model 1, 2DS5 arose in the centromeric site, 2DS3 in the telomeric site, whereas in model 2, 2DS3 arose in the centromeric site, 2DS5 in the telomeric site. Subsequent recombination between variants in the centromeric and telomeric sites gave rise to the variants observed in the modern KIR haplotypes. Model 2 requires only two such recombinations and is more parsimonious than model 1, which requires three. However, the greater diversity observed in *2DL5B-2DS5* than *2DL5A-2DS5*, favors model 1 over model 2.

We aligned the seven sequences of genomic segments containing *2DL5A or 2DL5B* and the neighboring *2DS3* or *2DS5* (Figure 5A), and performed domain-by-domain phylogenetic analysis with construction of neighbor-joining and parsimony trees. Domains were defined as individual introns or exons, recombination breakpoints were determined both by visual inspection of the alignment and use of RDP. The results showed that these segments divide into three parts having different evolutionary histories (Figure 5B). Sequences of the region extending from 5′ of the *2DL5* start codon through intron 2 of *2DL5*, formed two groups corresponding to *2DL5A* and *2DL5B*. In this 1.5 kb region, 2DL5B*00601 is divergent, differing by 21 unique substitutions from other *2DL5* and other *KIR*. In the proximal region, extending from exon 3 of *2DL5* through to intron 6 of *2DS3* or *2DS5*, the sequences form two groups, corresponding to the presence of either *2DS3* or *2DS5*. This grouping is independent of the genomic location. Analysis of the region extending from intron 6 to the end of the *2DS3* or *2DS5* gene showed that 2DS3*00103 forms an outgroup to the other *2DS3 2DS5* variants. In the latter group, three substitutions distinguish sequences derived from the centromeric and telomeric regions.

Taken together, these data are consistent with two alternative models for the evolution of the segments containing *2DL5* and either *2DS3* or *2DS5* (Figure 5C). Common to both models, the progenitor haplotype had *2DL5*, and the common ancestor of *2DS3* and *2DS5*, in the centromeric part of the *KIR* locus. This proposition is supported by presence in the centromeric region, and absence from the telomeric region, of chimpanzee and orangutan *2DL5* orthologs. Subsequently this segment duplicated, with transposition of the daughter locus to the telomeric region. These two segments then diverged independently to form *2DS3* and *2DS5*, which now differ by ∼250 nucleotide substitutions in 15kb. Subsequent recombination and/or gene conversion events led to *2DS3* and *2DS5* being present in both the centromeric and telomeric locations. The two models differ in the locations where *2DS3* and *2DS5* arose (Figure 5C).

Model 1 proposes that *2DS5* arose in the centromeric region and *2DS3* arose in the telomeric region; in contrast, model 2 proposes that *2DS3* arose in the centromeric region and *2DS5* arose in the telomeric region (Figure 5C). Model 1 requires three recombination events to generate the haplotypes present in the panel (Figure 5C left), the two proposed above and an additional recombination within the *Tel* segment. Supporting model 1 is the greater diversity of *2DS5* in the centromeric location, suggesting that *2DS5* has existed there for the longer period of time. Alternatively, the increased *2DS5* diversity may not be a consequence of age, but of recent selection. Model 2 is the more parsimonious model, because it requires only two recombination events to generate the haplotypes seen in the panel (Figure 5C, right). In summary, there is no compelling evidence for ruling out either of the two models. What is clear, however, is that genetic diversity arising in the two locations now containing *2DS3* and *2DS5* genes has subsequently been moved between them by recombination.

### The centromeric and telomeric motifs of *A* and *B* haplotypes diverged at very different times

**Figure 6:**
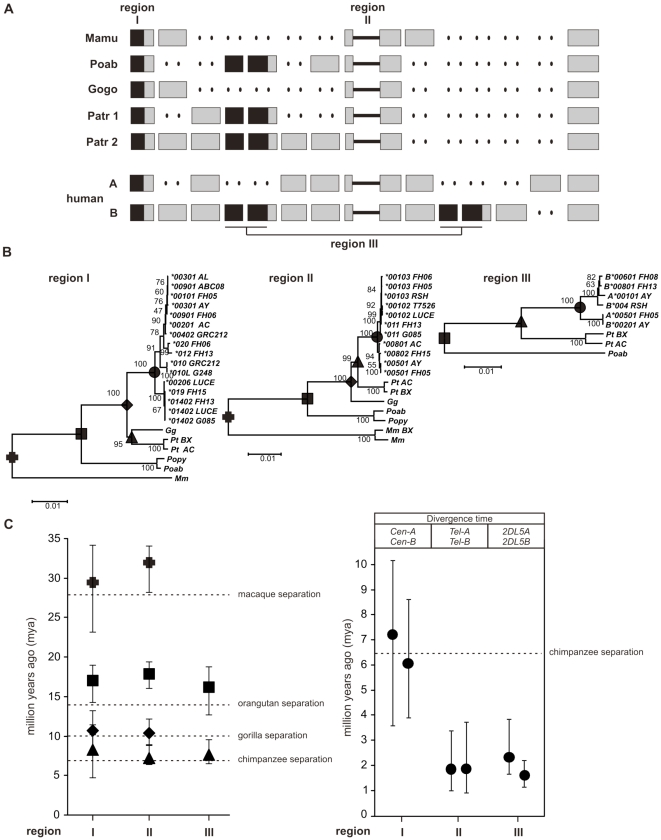
The *Cen-A* and *Cen-B* difference arose long before that between *Tel-A* and *Tel-B*. Three genomic segments with orthologs or paralogs in other higher primates were chosen for divergence time estimation. (A) Shows the three regions used for the analysis and the haplotypes from which they were obtained (human (AC006293/AC011501, AL133414, AY320039, and this study) chimpanzee (BX842589, AC155174), gorilla (CU92894), orangutan (EF014479, AC200148) and rhesus macaque (BX842590, BX842591)). They comprise region I, a 5.5 kb segment including the 5′ part of *3DL3* excluding intron 1, a 14 kb segment from the intergenic region between *3DP1* and *KIR2DL4* (region II), and a 16.8 segment beginning in intron 3 of *2DL5A/B* extending into the neighboring *2DS3*/5 gene (region III). For the latter, both the centromeric and telomeric variants were included in the analysis. (B) Shows the phylogenetic trees for regions I, II and III, with bootstrap values for the nodes. The nodes denoted by dark shaded symbols were those used for divergence time estimates. (C) Plotted here are the divergence time estimates using the same symbols as in panel B. For comparison, the dotted horizontal lines indicate the lower limits for divergence times from the human lineage of rhesus macaque, orangutan, gorilla and chimpanzee as assessed from the fossil record [73]. The left panel examines the divergence of human KIR from KIR in other primates, the right panel examines the divergence of different forms of human KIR and the results from two independent runs of the program are shown. Analysis of region I estimates the divergence time for *Cen-A* and *Cen-B*, whereas analysis of region II estimates the divergence of *Tel-A* and *Tel-B*, and analysis of region III estimates the time of duplication for *2DL5A* and *2DL5B* as well as *2DS3* and *2DS5*.

As all modern human populations have *A* and *B* haplotypes, whereas this distinction has not been described in other primates, it was of interest to know when and how the *A* and *B* haplotype motifs diverged from a common ancestor. To estimate divergence times, we performed phylogenetic analysis on three potentially informative regions, shown in black in Figure 6A. Region I is a 5.5 kb segment of the 3DL3 gene that starts 250 bp upstream of the start codon and ends 400 bp 5′ of exon 5 at the recombination breakpoint in the orangutan sequence [42]; Region II comprises the 14 kb intergenic region between *3DP1* and *2DL4*. Region III is a 16.8 kb segment beginning 100 bp 5′ of exon 3 of the *2DL5* gene and extending to 500 bp 3′ of exon 5 of the neighbouring *2DS3* or *2DS5* gene, including the intergenic region between them.

Phylogenetic analysis was performed on datasets that combined orthologous/paralogous sequences from human and other primate species, and from which recombinant sequences were excluded. Regions I and II have counterparts in rhesus macaque, orangutan, gorilla, and chimpanzee, whereas region III is limited to humans and great apes. In the phylogenetic trees, the deeper branches segregate the *KIR* from different species (Figure 6B). The positions of these deeper branch points (nodes) were used to calculate times for the divergence of humans from macaque, orangutan, gorilla and chimpanzee. These divergence times, plotted in Figure 6B, correspond well with estimates based on the fossil record, giving confidence in the validity of the analysis.

The more recent branch points in the phylogenetic trees distinguish groups of human sequences and mark events in *KIR* locus evolution that occurred in the human lineage since separation from the chimpanzee lineage. The two main lineages in region I, correspond to *3DL3* alleles that segregate with the *Cen-A* and *Cen-B* motifs. Their divergence time is estimated to be 6-7.2 million years ago, contemporaneous with the divergence time of the human and chimpanzee lineages (Figure 6C). Further supporting this conclusion, the chimpanzee and gorilla genes form a distinct branch of the tree (Fig. 6B), suggesting that several *Cen* lineages were present at the time of separation of humans from chimpanzees, whereupon chimpanzees retained a lineage distinct from those present in modern day humans. This analysis clearly shows that *Cen-A* and *Cen-B* motifs have been present throughout much, if not all, of human evolution.

The two main lineages in region II, the intergenic region between *3DP1* and *2DL4*, segregate with the *Tel-A* and *Tel-B* motifs. Their estimated divergence time is ∼1.7 million years ago (Figure 6C), several million years after the human-chimpanzee separation, but before the estimated emergence of the modern human species (150,000-190,00 years ago, [43]). The two main lineages in region III represent the duplication of *2DL5* and the ancestor of *2DS3* and *2DS5*, and their divergence in the centromeric and telomeric regions of the *KIR* locus. The estimated divergence time, and thus the time of the duplication event, is also estimated at ∼ 1.7 million years ago. These independent analyses of a non-coding intergenic region, and of a segment carrying two characteristic *B* haplotype genes are concordant in showing that the *Cen-A*/*Cen-B* dichotomy existed for several million years before emergence of the *Tel-A*/*Tel-B* dichotomy. The results also indicate that all four of the *KIR* gene motifs (*Cen-A*, *Cen-B*, *Tel-A*, and *Tel-B*) have been present throughout the evolution of the modern human species.

## Discussion

The distinctive organization of the human *KIR* locus drives the generation of gene-content diversity. Conserved genes are situated at the middle (*3DP1* and *2DL4*) and ends (*3DL3* and *3DL2*) of the locus, creating a framework around two regions of variability, in which highly homologous *KIR* genes are packed close together in head-to-tail configuration and separated by short and highly conserved intergenic regions [33]. These properties have facilitated the numerous asymmetric recombinations that duplicated *KIR* genes, deleted *KIR* genes, and formed new hybrid *KIR* genes with novel ligand-binding and signaling functions [44]. The propensity for recombination was further appreciated from comparison of humans with apes and monkeys, from which the conserved framework was further reduced to the extremities of the locus –the 5′ part of *3DL3* and the 3′ part of 3DL2 – plus part of *3DP1* but all of *2DL4* in the central region [42]. The only site of unique sequence in the *KIR* locus is in the 14kb intergenic region that separates *3DP1* from *2DL4* and divides the locus into centromeric and telomeric parts of similar size [45]. This unique sequence has been the site for events of reciprocal recombination that allowed centromeric and telomeric gene-content motifs to reassort in different combinations and form new variant *KIR* haplotypes [34], [46]. That seven of the eight possible combinations of four common centromeric and two telomeric motifs are represented in the 27 *KIR* haplotypes studied here, testifies to the importance of this mechanism.

Well represented in the *KIR* haplotype panel are two highly divergent gene-content haplotypes. One of these is the ‘long’ cB01|tB01 haplotype [25] that contains all *KIR* genes except *2DS4*, and has all the *B* haplotype specific genes and alleles. The second is the *A* haplotype (cA01|tA01) that has 2DS4 and none of the B haplotype specific genes and alleles. The extent of the differences between these two haplotypes is further emphasized at the allele level, because no allele for any gene is held in common. The other five gene content haplotypes are all *B* haplotypes that lack some of the *B*-specific genes, either as a consequence of deletion (eg: cB02|tB01), or recombination that introduced either the centromeric (eg: cA01|tB01) or telomeric (eg: cB01|tA01) motif of the *A* haplotype. From epidemiological studies, a variety of disease associations have been made with differences between *A* and *B* haplotypes [47]. The prevalence of natural recombinants should allow further examination to test the contributions of centromeric and telomeric *A* and *B* motifs to these associations.

Natural division of *KIR* haplotypes, and their constituent centromeric and telomeric motifs, into two distinctive groups appears to be unique to the human species. Moreover, a mixture of *A* and *B* haplotypes is present in all human populations (N>150) that have been genotyped for *KIR*. Thus the combination of *A* and *B* haplotypes appears to confer selective advantage, as is most clearly illustrated by the dominance and equal frequency of two, maximally divergent *KIR* haplotypes (one *A* and one *B*) in the Yucpa population of Venezuelan Amerindians [24], [48]. From phylogenetic comparisons, we have been able to explore the evolution of the *A* and *B* haplotypes and the differences between them. Of the genes typifying either *A* or *B* haplotypes, only *2DS4* and *2DL5*, respectively, are present in chimpanzee, but in that species they are usually found linked on the same haplotypes in the centromeric region, the region containing all chimpanzee *KIR* except the *2DL4* and *3DL* framework genes (L. Abi-Rached, manuscript in preparation). In human *KIR* haplotypes, a comparable number of genes are found in the centromeric (N = 8) and telomeric (N = 7) regions, indicating that recent gene expansion in the telomeric part of the *KIR* locus is specific to the human lineage. As part of that expansion both *2DS4* and *2DL5* were moved into the telomeric region, but as part of different and mutually exclusive gene-content motifs. Consistent with the view that colonization of the telomeric region with lineage Ib and lineage III genes is human specific, the telomeric region of orangutan KIR haplotypes comprises only the framework genes.

**Figure 7:**
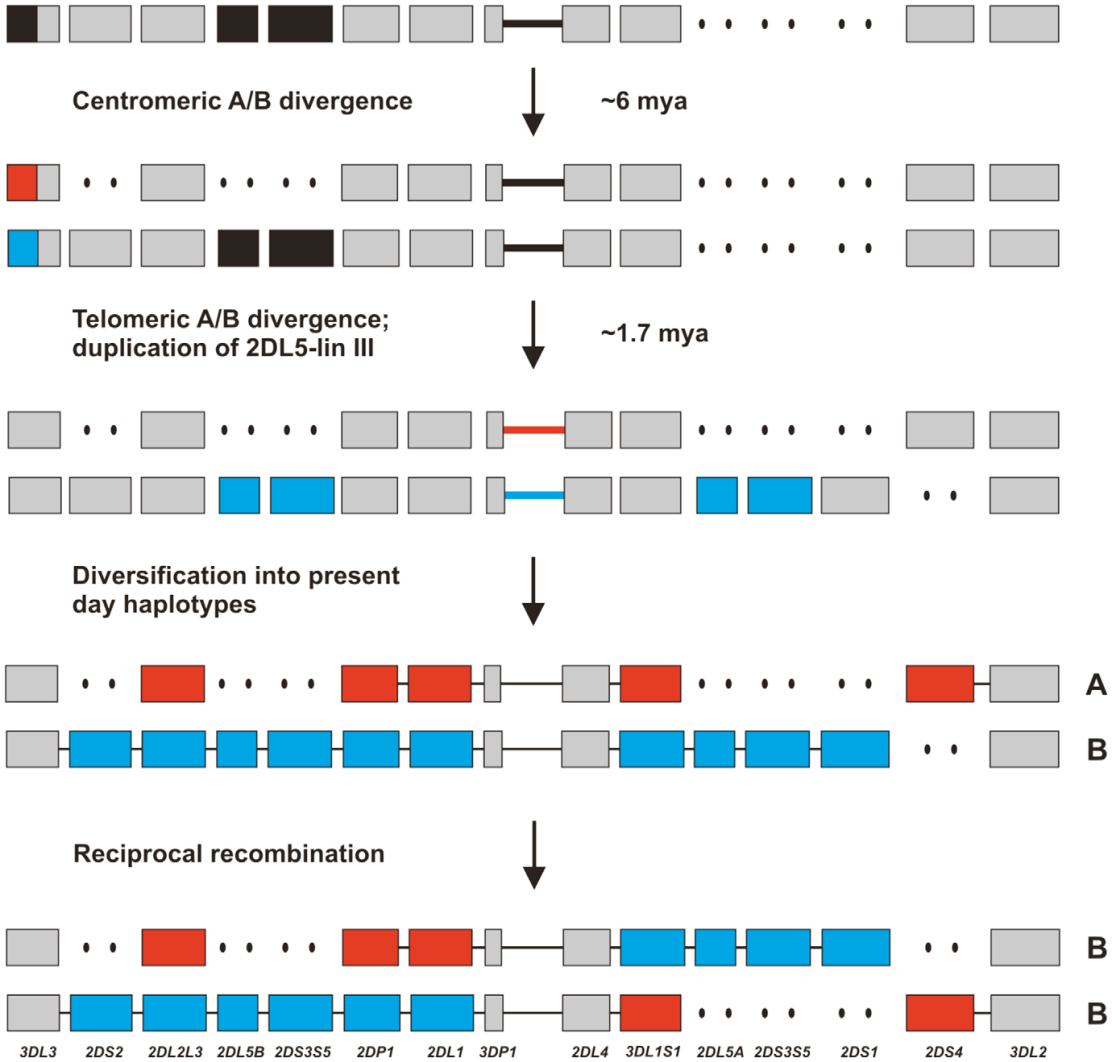
Model for the evolution of the A and B haplotypes. The progenitor haplotype contained the *2DL5-lineage III* segment in the centromeric region. The diversification that resulted in the formation of the *Cen-A* and *Cen-B* lineages occurred ∼6 mya, contemporaneous with human:chimpanzee speciation. The *Tel-A:Tel-B* divergence is much younger occurring ∼1.7 mya conincident with the timing for the duplication of the *2DL5-lineage III* progenitor that gave rise to *2DL5-2DS3* and *2DL5-2DS5*. This duplication could have produced either a *Cen-B:Tel-B* or *Cen-A:Tel-B* haplotype. Subsequent recombinations have given rise to the haplotype structures present today.

Our phylogenetic and divergence-time analyses indicate that evolution of the *A*, *B* haplotype difference occurred in the centromeric region around the time of the human-chimpanzee separation some 6 million years ago. As *2DL5* is present on ∼50% of human and chimpanzee KIR haplotypes, it is likely that the common ancestor also had both *2DL5^+^* and *2DL5^−^* haplotypes. This difference could have provided the foundation on which the *Cen-A*/*Cen-B* difference was built (Figure 7). Leading to the more recent evolution of the *Tel-A/Tel-B* difference was duplication of *2DL5* and its neighboring lineage III *2DS*, with movement of one of daughter pair to the telomeric region. The difference between *Tel-A* and *Tel-B*, as assessed from the time of duplication of 2DL5, emerged ∼1.7 million years ago, >4 million years after the *Cen-A/CenB* difference (Fig. 6) and >1 million years before the origin of the modern human species, in the last 200,000 years [43]. Another key event in forming the *Tel-A/Tel-B* difference was movement of *2DS4* from a centromeric location (as in chimpanzee *KIR* haplotypes) to the telomeric location observed in human KIR haplotypes. Because of these species-specific locations, we could not calculate when these two forms diverged and thus distinguish between the following two possibilities: the first model is that *2DS4* moved from centromeric to telomeric location specifically on the human lineage; the second, that both types of *2DS4* **^+^** haplotypes existed in the common ancestor, but those containing centromeric *2DS4* were lost on the human lineage, whereas haplotypes containing telomeric *2DS4* were lost on the chimpanzee lineage. Either way, the establishment of alternative gene content motifs in both the centromeric and telomeric regions, provided the basic structure within which increasingly elaborate *A* and *B* motifs could evolve. A key finding from this analysis is that the *A/B* haplotype dichotomy had been long in place when modern humans emerged and has existed throughout their evolution, consistent with the presence of *A* and *B* haplotypes in all modern human populations. NK cells play essential roles in both immune defense and reproduction, physiological functions that are essential for the survival of populations and species [47], [49], [50], [51]. Because *A KIR* haplotypes favor elimination of infection [7], whereas *B KIR* favor reproductive success [18], a simple model is that balancing selection on the *A* and *B* haplotypes stems from the *A* haplotypes having evolved under pressure on the immune system, whereas the *B* haplotypes evolved under pressure on the reproductive system [48].

With the result of this study, 12 *A KIR* haplotypes and 15 *B KIR* haplotypes have been defined at the highest level of resolution. The gene-content, and allele-content motifs contained in the centromeric and telomeric regions of these 27 haplotypes are likely to account for many common haplotypes in many human populations, and should provide a strong base from which to investigate the rarer and more population-specific haplotypes. Several of these have already been investigated and include ones in which framework genes are duplicated [20], [35], [52], [53] deleted [21], [54] or fused with another gene to collapse the telomeric region [20]. There is increasing evidence that taking account of the immunogenetics of KIR can improve the outcome of allogeneic hematopoietic cell transplantation as therapy for acute myelogenous leukemia [16], [55], [56], [57]. The results of this study will further the practical application of KIR genotyping to this valuable clinical goal.

## Materials and Methods

### Cell lines/source DNA

The DNA used for library construction was extracted from a panel of cell lines chosen to encompass major ethnic groups. In addition, the cell lines chosen were inferred to have both and *A* and *B KIR* haplotype and to contain representatives of the most common *B* haplotypes (Table 3). DNA was prepared from B-LCLs using a Qiagen (Valencia, CA) genomic DNA extraction kit according to the manufacturers instructions. DNAs forming a diversity panel of 48 each of Caucasian, Asian, African-American, and Hispanic were obtained from the Research Cell Bank (RCB) at the Fred Hutchinson Cancer Research Center and are commercially available (File S1).

**Table 3:** Fosmid clone resources.

Source	Ethnicity	Library density(colonies/ml)	Host Cell
ABC08	Yoruba	a	a
FH05	Caucasian	2500	EPI300
FH06	Hispanic	2170	EPI300
FH08	American-Black	1500/1800	XL1_B/EPI300
FH13	American-Black	1500/1800	XL1_B/EPI300
FH15	Caucasian	1640	XL1_B
G085	Spanish Gypsy	3800	EPI300
G248	unknown	a	a
GRC212	Guarani South Amerindian	3000	XL1_B
LUCE	unknown	3600	EPI300
RSH	South African Zulu	3500	EPI300
T7526	Chinese	3850	EPI300

a clones were obtained from end sequenced fosmid libraries described in [59].

### Fosmid libraries, cloning, typing, sequencing

Fosmid Isolation was carried out as described in Raymond *et al*. [58] with modifications. Library constructions used the Epicentre copy-control vector pCC1. Sheared, end-repaired inserts were size-selected to be 30–50 kbp by pulsed-field-gel electrophoresis. To produce 10^6^-clone libraries we typically started with 20 µg of genomic DNA. Packaging was carried out with the Epicentre MaxPlax extracts and transfection was into Epicentre EPI300-T1^R^ *E. coli* cells. This cloning system allows induction of the fosmid copy number to ∼50 copies/cell, which was critical to simplification of library screening and downstream sequencing. The high-copy-number origin was induced when growing saturated cultures from replicas of the master-library plates; lysed aliquots of induced cultures were used directly as a source of PCR template during the STS-content mapping of 3000-fosmid pools and at all subsequent stages of screening. The 3000-clone pool size was optimal as we found more complex pools reduced the number of PCR assays required for STS-content mapping, but they also decreased the reliability of initial tiling-path choices (by increasing the density of positive wells on the plate) which increased the work involved in finding the positive clone in the pool. Fosmids were derived from libraries ABC8 and G248 by computational screening of end sequences as described [59].

#### STS design

For KIR screening we used a panel of unlabeled primers designed locally and ordered from generic vendors that amplified each of the known KIR genes. File S1 includes the sequences and gene specificity for the primer pairs used for all of the fosmid screening carried out in this report. Samples are scored as positive or negative for a particular PCR assay based on SYBR green fluorescence. In some cases when allele-specific information was required, the PCR products were sequenced. Data were collected on an ABI 7900 instrument, operating in real-time (as opposed to end-point) mode. With 3000-clone pools, single-clone isolation proceeded in two steps: preparation and screening of 100-clone subpools. Typically, a single 384-well plate of subpools were prepared from each positive 3000-clone pool. A single positive subpool was then plated for single colonies, ∼1000 of which were picked robotically to produce cultures employed for the final stage of screening. One positive-single-colony isolate was chosen for re-streaking, re-testing, and subsequent characterization.

#### Sequencing

Fosmids were sequenced using shotgun-sequencing protocols as outlined previously [60]. Steps included (1) subcloning of target DNA into pUC19 after shearing to ∼4 kbp with a Genomic Solutions Hydroshear instrument, size-selection to 2–7 kbp on an agarose gel, and end repair using a mixture of T4 DNA polymerase and the Klenow fragment of DNA polymerase I; (2) robotic colony picking, growth of a saturated culture, and chemical lysis of a 1-µl aliquot of culture; (3) amplification of released DNA with the Amersham TempliPhi reagent; (4) thermocycling dideoxy sequencing reactions based on ABI BigDye chemistry; (5) separating the products on ABI 3730xl capillary-sequencing instruments; (6) assembling sequencing reads with the phred/phrap system; (7) carrying out one round of finishing using software to support primer design (average 1 finishing read per 2 fosmids). PCR-resequencing was currently carried out according to established procedures [61], [62]. All data from PCR-resequencing assays were automatically interpreted by software developed in house (GeMS), which accurately interprets sequence traces from heterozygous DNAs [62], [63].

Sequences have been deposited in GenBank with the following accession numbers: FH05_A_hap  =  GU182338; FH05_B_hap  =  GU182339; FH06_A_hap  =  GU182340; FH06_BA1_hap - GU182341; FH08_A_hap  =  GU182342; FH08_BAX_hap  =  GU182343; FH13_A_hap  =  GU182344; FH13_BA2_hap  =  GU182345; FH15_A_hap  =  GU182346; FH15_B_hap  =  GU182347; G085_A_hap  =  GU182348; G085_BA1_hap  =  GU182349; G248_Ahap  =  GU182350; G248_BA2hap  =  GU182351; GRC212_AB_hap  =  GU182352; GRC212_BA1_hap  =  GU182353; LUCE_A_hap  =  GU182354; LUCE_Bdel_hap  =  GU182355; RSH_A_hap  =  GU182356; RSH_BA2_hap  =  GU182357; T7526_A_hap  =  GU182358; T7526_Bdel_hap  =  GU182359; ABC08_A1hap  =  GU182360; ABC08_AB_hap_central_partial  =  GU182361; ABC08_AB_hap_telomere_partial GU182362. Alleles and haplotypes have been named according to the guidelines established by the KIR Nomenclature Committee [64] and deposited into IPD-KIR (http://www.ebi.ac.uk/ipd/kir/).

### Computational analyses

#### Phylogenetic analysis

The dataset included the 24 newly sequenced haplotypes and the three human haplotypes deposited in GenBank (AC006293/AC011501, AL133414, AY320039) [32], [33], [34]. For the divergence time estimation chimpanzee (BX842589, AC155174) [65], gorilla (CU92894), orangutan (EF014479, AC200148) [42] and rhesus macaque (BX842590, BX842591) [65] sequences were used. Sequences of the individual genes were aligned using CLUSTAL X [66] or MAFFT [67] and manually corrected in BIOEDIT (http://www.mbio.ncsu.edu/BioEdit/bioedit.html). The alignment was then divided into domains generally following intron-exon boundaries, except for intron 6 which was divided further into 3 regions. The first of these (intron 6a) starts at the beginning of the intron and ends at the beginning of the deletion common to *MmKIR3DL1* and *MmKIR3DL10* (approx. 750 bp), the second (intron 6b) begins here and ends at the beginning of the LINE insertion common to *KIR3DL2* and *PtKIR3DL1/2* (approx. 2.9 kb) and the third (intron 6c) starts after the LINE insertion and ends at the end of the intron (approx. 600 bp). Each of these alignments was used for neighbor-joining (NJ) and parsimony analyses. The NJ analysis was performed using MEGA version 4 (http://www.megasoftware.net/) [68] with 500 replicates, pairwise deletion, midpoint rooting, and the Tamura-Nei method. PAUP*4.0b10 (http://paup.csit.fsu.edu/) and the tree bisection-reconnection branch-swapping algorithm were used for parsimony analyses with 500 replicates and a heuristic search. Comparison of the resulting trees revealed no differences. Only neighbor-joining trees are presented in the figures.

#### Nucleotide diversity

The average number of nucleotide differences per site between two sequences, or nucleotide diversity, Pi, and its sampling variance and standard error [69] were calculated using DNASP (http://www.ub.es/dnasp/) [38]. Genes were examined individually and framework genes were divided based on their location on either *A* or *B* segments. Also, *2DL1* and *2DP1* were analyzed separately according to their presence on *Cen-A* vs. *Cen-B* segments. Finally, the *2DL5* sequences were subgrouped according to their linkage to either *2DS3* or *2DS5* and their presence in either the centromeric or telomeric interval.

#### Divergence time estimation

Divergence time estimation was completed using MCMCTREE in the PAML package [70], [71]. Starting kappa and alpha values were estimated using baseml in the PAML package [70], [71] and rgene was estimated from neighbor joining trees from MEGA [68]. Three datasets including both human and non-human primate sequences were analyzed, a 5.5 kb segment extending from 250 bp 5′ of the start codon to 450 bp 3′ of exon 5 (excluding intron 1) of *KIR3DL3*, a 14 kb segment from the region between *3DP1* and *KIR2DL4*, and a 16.9 kb segment beginning 100bp 5′ of exon 3 of *KIR2DL5* extending to 550 bp 3′ of exon 5 of the neighboring lineage III gene. Each dataset was analyzed for recombinants using RDP [72] and recombinants were excluded from the analysis. NJ trees were constructed in MEGA and parsimony trees were constructed in PAUP as described above. The calibration times used for the analysis were human-chimpanzee split 6.5-10 mya, gorilla speciation >10 mya, orangutan speciation <18 mya, and rhesus macaque speciation 23-34 mya [73]. Datasets and control files are available upon request to the authors.

## Supporting Information

File S1 This file includes data arranged in three tables, including supplementary Table 1 - KIR haplotype summary statistics, supplementary, Table 2 - KIR gene PCR-SSP for library screening and haplotyping, and supplementary Table 3 - KIR diversity cell panel.
(DOC)
