# Supplementary material for: Different Patterns of Evolution in the Centromeric and Telomeric Regions of Group A and B Haplotypes of the Human Killer Cell Ig-Like Receptor Locus
Source: PLoS One. 2010 Dec 29;5(12):e15115. doi: 10.1371/journal.pone.0015115 (PMC3012066; doi:10.1371/journal.pone.0015115)
Supplement: File S1 — This file includes data arranged in three tables, including supplementary Table 1 - KIR haplotype summary statistics, supplementary, Table 2 - KIR gene PCR-SSP for library screening and haplotyping, and supplementary Table 3 - KIR diversity cell panel. (DOC) [file pone.0015115.s001.doc]

**Supplementary Table 1. KIR haplotype summary statistics**

|  | **KIR-Haplotype** | | | | | | |
| --- | --- | --- | --- | --- | --- | --- | --- |
|  | **cA01|tA01** | **cB02|tA01** | **cB01|tA01** | **cB02|tB01** | **cB01|tB01** | **cA01|tB01** | **cB03|tB01** |
| **Number** | **12** | **3** | **3** | **3** | **3** | **2** | **1** |
| **length (bp)** | 142264 | 126815 | 185828 | 156120 | 213851 | 169453 | 169519 |
| **Nucleotide diversity (Pi)#** | 2.1 | 2.8 | 4.3 | 2.0 | 1.1 | N.A | N.A |
| **S.D (Pi)#** | 0.4 | 0.8 | 1.4 | 0.8 | 0.5 | N.A | N.A |
|  |  |  |  |  |  |  |  |
| **Neutrality Tests** |  |  |  |  |  |  |  |
| **Tajima's D** | -0.7 | N.A | N.A | N.A | N.A | N.A | N.A |
| **Fu and Li's D*** | -0.6 | N.A | N.A | N.A | N.A | N.A | N.A |
| **Fu and Li's F*** | -0.7 | N.A | N.A | N.A | N.A | N.A | N.A |

|  | **KIR-motif** | | | | |
| --- | --- | --- | --- | --- | --- |
|  | **cA01** | **cB01** | **cB02** | **tA01** | **tB01** |
| **Number** | **14** | **6** | **6** | **16** | **7** |
| **length (bp)** | 65646 | 110119 | 52179 | 63022 | 90268 |
| **Nucleotide diversity (Pi)#** | 1.6 | 3.2 | 1.2 | 3.3 | 2.1 |
| **S.D (Pi)#** | 0.3 | 1.1 | 0.2 | 0.5 | 0.4 |
|  |  |  |  |  |  |
| **Neutrality Tests** |  |  |  |  |  |
| **Tajima's D** | -0.5 | -0.1 | -0.3 | -0.3 | **2.0*** |
| **Fu and Li's D*** | 0.0 | 0.0 | -0.3 | -0.2 | 1.4 |
| **Fu and Li's F*** | -0.2 | 0.0 | -0.3 | -0.3 | **1.7*** |

***p<0.05 #; x10^3^**

**Supplementary Table 2. KIR gene PCR-SSP for library screening and haplotyping**

**(1) Forward primer**

| **Gene** | **Position** | **forward_primer_Sequences** |
| --- | --- | --- |
| 3DL3 (Exon 4) | 361-383 | CACAGAAAACCTTCCCTCCTGGC |
| 2DS2 (Exon4) | 168-197 | GCACTTCCTTCTGCACAGAGAGGGGAA**C**TA **(original; G)** |
| 2DL3 (Exon7) | 716-735 | **CTTCCAG**GTAACCCCAGACACCTGCAT |
| 2DL2 (Exon4) | 91-110 | CTCCTGGCCCACCCAGG**A**CG **(original; T)** |
| 2DL5 (Exon 8-9) | 820-838 | GACCAAGAGCCTGC**G**GGGG **(original; C)** |
| 2DL5.2 (intron 6) | intron6 | TGTAAATCAATATCTGGCAGAGGAGTG**G**TA **(original; A)** |
| 2DL5.1 (intron 6) | intron6 | AATCAATATCTGGCAGAGGAGTG**G**TT **(original; A)** |
| 2DS3 (Exon 4) | 205-230 | ACTTTGCGCCTCATTGGAGAGCACAT |
| 2DS3/5 (Exon4) | 211-230 | CGCCTCATTGGAGAGCACAT |
| 2DP1 (Exon 4) | 358-380 | CACAGAAAACCTTCCCTCCTGGC |
| 2DP1_hpltA (Exon 4) | 358-380 | CACAGAAAACCTTCCCTCCTGGC |
| 2DP1_hpltB (Exon 4) | 457-477 | CTTCTGCACAGAGAGGGG**C**CA **(original; A)** |
| 2DL1 (Exon4) | 241-262 | TCCAAGGCCAACTTCTCCA**A**CA **(original; T)** |
| 3DP1 (Exon 4) | 462-486 | TCTGCACAGAGAGGGGAAGTTT**C**AT **(original; A)** |
| 2DL4A (Exon5) | 389-406 | CGGGCCCCACGGTTC**A**CA **(original; G)** |
| 2DL4B (Exon5) | 390-406 | GGGCCCCACGGTTC**A**CG **(original; G)** |
| 3DL1A (Exon 3) | 96-116 | TGCCTGGCCCAGCGCTGTGGT |
| 3DL1B (Exon 3) | 96-116 | TGCCTGGCCCAGCGCTGTGGT |
| 2DS5 (Exon 4) | 208-230 | TTGCGCCTCATTGGAGAGCACAT |
| 3DS1 (Exon 8-9) | 1125-1147 | AAGAGCCTGCAGGGAACAGAAGT |
| 2DS4L (Exon 5) | 458-484 | GCTCCTATGACATGTACCATCTATCCA |
| 2DS4S (Exon 5) | 440-462 | TGTCCTGCAGCTCCATCTATCCA |
| 2DS1 (Exon4) | 248-272 | CCAACTTCTCCATCAGTCGCAT**C**AA **(original; G)** |
| 3DL2 (Exon 5) | 692-710 | GCCCCACGGTTCAGGCAGG |

**(2) Reverse Primer**

| **Gene** | **Position** | **reverse_primer_Sequences** |
| --- | --- | --- |
| 3DL3 (Exon 4) | 557-576 | GGTCCCTGCAAGGGCA**T**GTG **(original; G)** |
| 2DS2 (Exon4) | 337-358 | TGTCCAGAGGGTCACTGGGAGC |
| 2DL3 (Exon7) | 809-821 | **CTCTGCTTCGTAAGACTTAC**TTTTTTTGT**C**GC **(original; T)** |
| 2DL2 (Exon4) | 266-286 | CTGCAAGGTCTTGCATCA**C**GG **(original; T)** |
| 2DL5 (Exon 8-9) | 1011-1034 | TGCTTATGGGCAGGAGACAATGAT |
| 2DL5.2 (intron 6) | intron6 | GACCCCGCCAAACCTCACG |
| 2DL5.1 (intron 6) | intron6 | GACCCCGCCAAACCTCACG |
| 2DS3 (Exon 4) | 333-361 | CGATGTCCAGAGGGTCACTGGGAGCT**C**AA **(original; G)** |
| 2DS3/5 (Exon4) | 337-358 | TGTCCAGAGGGTCACTGGGAGC |
| 2DP1 (Exon 4) | 606-627 | CACTGGGAGCTGACAACTG**C**TG **(original; A)** |
| 2DP1_hpltA (Exon 4) | 522-544 | CGATGGAGAAGTTGGCCTTG**T**AA **(original; G)** |
| 2DP1_hpltB (Exon 4) | 618-639 | TGTCCAGAGGGTCACTGGGAGC |
| 2DL1 (Exon4) | 331-353 | AGAGGGTCACTGGGAGCTGA**A**AC **(original; C)** |
| 3DP1 (Exon 4) | 622-643 | TGTCCAGAGGGTCACTGGGAGC |
| 2DL4A (Exon5) | 541-559 | GGGTGGCAGGACCCAGAGG |
| 2DL4B (Exon5) | 541-559 | GGGTGGCAGGACCCAGAGG |
| 3DL1A (Exon 3) | 223-249 | GCTCATGTTGAAGCTCTCCTGGAA**A**AA **(original; T)** |
| 3DL1B (Exon 3) | 202-228 | GAATATTCTGCCATGGAAGATGGG**G**AT **(original; A)** |
| 2DS5 (Exon 4) | 333-354 | CAGAGGGTCACTGGGCGCT**C**AC **(original; G)** |
| 3DS1 (Exon 8-9) | 1219-1240 | GGGCGAGTGATTTTTCTCTGTGTGA |
| 2DS4L (Exon 5) | 613-634 | AGTTTGACCACTCGTAGGG**G**GC **(original; A)** |
| 2DS4S (Exon 5) | 613-634 | AGTTTGACCACTCGTAGGG**G**GC **(original; A)** |
| 2DS1 (Exon4) | 331-352 | GAGGGTCACTGGGAGCTGA**G**AA **(original; C)** |
| 3DL2 (Exon 5) | 896-913 | ACCACACGCAGGGCAGGG |

**Red_charater;** Sequence modification for specificity on PCR

Blue_character; Original Sequence

**Green_character; Intron sequence**

**B/BA1/Bdel hap specific SSP**

|  | **Forward_primer** |  |
| --- | --- | --- |
| **B_hap specific** | B_4850_del_F | GATGATGAAGATGAAGATAGATAAT |
|  |  |  |
| **BA1_hap specific** | BA1/Bdel_5010_G_F | TAGAGAATTTGTAGATAGGCACGAG |
|  |  |  |
| **Bdel_hap_specific** | BA1/Bdel_5010_G_F | TAGAGAATTTGTAGATAGGCACGAG |
|  |  |  |
|  | **Reverse_primer** |  |
| **B_hap specific** | 2DL4/3DS1_2540_A_R | ACCATACGATAGTGTATGAAAAGAT |
|  |  |  |
| **BA1_hap specific** | 2DL4/3DL1_2540_G_R | ACCATACGATAGTGTATGAAAAGAC |
|  |  |  |
| **Bdel_hap_specific** | 2DL4/3DS1_2540_A_R | ACCATACGATAGTGTATGAAAAGAT |

**Supplementary Table 3.** KIR diversity cell panel

| **Name** | **Ethnic** | **Name** | **Ethnic** | **Name** | **Ethnic** | **Name** | **Ethnic** |
| --- | --- | --- | --- | --- | --- | --- | --- |
| IHW30001 | AFA | HIP02143 | ASI | HIP09419 | HIS | HIP09152 | CAU |
| IHW30002 | AFA | HIP02553 | ASI | HIP09436 | HIS | HIP00597 | CAU |
| IHW30010 | AFA | HIP09423 | ASI | HIP09398 | HIS | HIP08709 | CAU |
| IHW30011 | AFA | HIP09404 | ASI | HIP09510 | HIS | HIP00228 | CAU |
| IHW30014 | AFA | HIP09415 | ASI | HIP09511 | HIS | HIP01370 | CAU |
| IHW30021 | AFA | HIP09410 | ASI | HIP09512 | HIS | HIP01435 | CAU |
| IHW30022 | AFA | HIP09403 | ASI | HIP09513 | HIS | HIP09189 | CAU |
| IHW30026 | AFA | HIP09447 | ASI | HIP09514 | HIS | HIP09208 | CAU |
| IHW30027 | AFA | HIP09548 | ASI | HIP09515 | HIS | HIP09102 | CAU |
| IHW30030 | AFA | HIP09452 | ASI | HIP09518 | HIS | HIP09105 | CAU |
| IHW30033 | AFA | HIP09453 | ASI | HIP09522 | HIS | HIP09209 | CAU |
| IHW30036 | AFA | HIP09564 | ASI | HIP09456 | HIS | HIP01428 | CAU |
| IHW30037 | AFA | HIP09484 | ASI | HIP09457 | HIS | HIP03171 | CAU |
| IHW30048 | AFA | HIP09489 | ASI | HIP09524 | HIS | HIP11455 | CAU |
| IHW30051 | AFA | HIP09490 | ASI | HIP09527 | HIS | HIP02774 | CAU |
| IHW30052 | AFA | HIP09493 | ASI | HIP09526 | HIS | HIP05438 | CAU |
| IHW30063 | AFA | HIP09495 | ASI | HIP09528 | HIS | HIP00133 | CAU |
| IHW30064 | AFA | HIP09497 | ASI | HIP09529 | HIS | HIP05018 | CAU |
| IHW30067 | AFA | HIP09485 | ASI | HIP09530 | HIS | HIP06999 | CAU |
| IHW30068 | AFA | HIP09575 | ASI | HIP09531 | HIS | HIP07659 | CAU |
| IHW30071 | AFA | HIP09684 | ASI | HIP09532 | HIS | HIP01397 | CAU |
| IHW30072 | AFA | HIP09543 | ASI | HIP09533 | HIS | HIP05796 | CAU |
| IHW30078 | AFA | HIP09501 | ASI | HIP09540 | HIS | HIP09188 | CAU |
| IHW30079 | AFA | HIP09625 | ASI | HIP09586 | HIS | HIP08889 | CAU |
| IHW30086 | AFA | HIP09774 | ASI | HIP09757 | HIS | HIP12479 | CAU |
| IHW30087 | AFA | HIP09620 | ASI | HIP09764 | HIS | HIP01062 | CAU |
| IHW30090 | AFA | HIP09777 | ASI | HIP09699 | HIS | HIP00973 | CAU |
| IHW30091 | AFA | HIP09778 | ASI | HIP09702 | HIS | HIP12478 | CAU |
| IHW30097 | AFA | HIP09781 | ASI | HIP09770 | HIS | HIP00686 | CAU |
| IHW30098 | AFA | HIP09785 | ASI | HIP09688 | HIS | HIP04906 | CAU |
| IHW30104 | AFA | HIP09787 | ASI | HIP04345 | HIS | HIP00521 | CAU |
| IHW30107 | AFA | HIP09791 | ASI | HIP05218 | HIS | HIP09147 | CAU |
| IHW30108 | AFA | HIP10146 | ASI | HIP04952 | HIS | HIP09927 | CAU |
| HIP09471 | AFA | HIP04055 | ASI | HIP01131 | HIS | HIP12195 | CAU |
| HIP03277 | AFA | HIP04484 | ASI | HIP01164 | HIS | HIP12655 | CAU |
| HIP02380 | AFA | HIP01158 | ASI | HIP00101 | HIS | HIP12481 | CAU |
| HIP09472 | AFA | HIP02554 | ASI | HIP01104 | HIS | HIP02106 | CAU |
| HIP03276 | AFA | HIP09408 | ASI | HIP09401 | HIS | HIP03149 | CAU |
| HIP09910 | AFA | HIP09411 | ASI | HIP09421 | HIS | HIP00123 | CAU |
| HIP02335 | AFA | HIP09416 | ASI | HIP09425 | HIS | HIP03189 | CAU |
| HIP01887 | AFA | HIP09435 | ASI | HIP09432 | HIS | HIP03399 | CAU |
| HIP02379 | AFA | HIP09445 | ASI | HIP09442 | HIS | HIP03682 | CAU |
| HIP09466 | AFA | HIP09486 | ASI | HIP09504 | HIS | HIP04971 | CAU |
| HIP09913 | AFA | HIP09491 | ASI | HIP09588 | HIS | HIP05478 | CAU |
| HIP09917 | AFA | HIP09494 | ASI | HIP09647 | HIS | HIP00003 | CAU |
| HIP09461 | AFA | HIP09496 | ASI | HIP09658 | HIS | HIP00012 | CAU |
| HIP09921 | AFA | HIP09498 | ASI | HIP09695 | HIS | HIP00083 | CAU |
| HIP09654 | AFA | HIP09788 | ASI | HIP09763 | HIS | HIP00087 | CAU |
